# Early Concepts in CT Image-Guided Robotic Vascular Surgery: The Displacement of Retroperitoneal Structures During Simulated Procedures in a Cadaveric Model

**DOI:** 10.3390/tomography11060060

**Published:** 2025-05-23

**Authors:** Balazs C. Lengyel, Ponraj Chinnadurai, Rebecca G. Barnes, Charudatta S. Bavare, Alan B. Lumsden

**Affiliations:** 1 Department of Cardiovascular Surgery, Houston Methodist Hospital, Houston, TX 77030, USA; 2 Department of Vascular and Endovascular Surgery, Semmelweis University, 1122 Budapest, Hungary; 3 The Bookout Center, Houston Methodist Academic Institute, Houston, TX 77030, USA

**Keywords:** image-guided surgery, vascular robotics, computer tomography, organ displacement, cadaveric model, laparoscopy

## Abstract

Background: CT image guidance and navigation, although routinely used in complex endovascular procedures, is an unexplored territory in evolving vascular robotic procedures. In robotic surgery, it promises the better localization of vasculature, the optimization of port placement, less inadvertent tissue damage, and increased patient safety during the dissection of retroperitoneal structures. However, unknown tissue displacement resulting from induced pneumoperitoneum and positional changes compared to the preoperative CT scan can pose significant limitations to the reliability of image guidance. We aimed to study the displacement of retroperitoneal organs and vasculature due to factors such as increased intra-abdominal pressure (IAP) due to CO_2_ insufflation and patient positioning (PP) using intraoperative CT imaging in a cadaveric model. Methods: A thawed, fresh-frozen human cadaveric model was positioned according to simulated procedural workflows. Intra-arterial, contrast-enhanced CT scans were performed after the insertion of four laparoscopic ports in the abdomen. CT scans were performed with 0–5–15–25 mmHg IAPs in supine, left lateral decubitus, right lateral decubitus, Trendelenburg, and reverse Trendelenburg positions. Euclidean distances between fixed anatomical bony and retroperitoneal vascular landmarks were measured and compared across different CT scans. Results: Comparing the effects of various IAPs to the baseline (zero IAP) in the same PP, an average displacement for retroperitoneal vascular landmarks ranged from 0.6 to 3.0 mm (SD 1.0–2.8 mm). When changing the PPs while maintaining the same IAP, the average displacement of the retroperitoneal vasculature ranged from 2.0 to 15.0 mm (SD 1.7–7.2 mm). Conclusions: Our preliminary imaging findings from a single cadaveric model suggest minimal (~3 mm maximum) target vasculature displacement in the retroperitoneum due to elevated IAP in supine position and higher displacement due to changes in patient positioning. Similar imaging studies are needed to quantify procedural workflow-specific and anatomy-specific deformation, which would be invaluable in developing and validating advanced tissue deformation models, facilitating the routine applicability and usefulness of CT image guidance for target delineation during robotic vascular procedures.

## 1. Introduction

Image-guided surgery (IGS) refers to a surgical approach where preoperative or intraoperative computer tomography is used to provide real-time, high-resolution, three-dimensional anatomical guidance during a procedure to enhance surgical precision and facilitate target localization.

When approaching IGS from a vascular surgical perspective, 3D imaging and image fusion guidance have been evolving with success in various complex endovascular procedures, including endovascular aortic repair and the treatment of endoleaks [1,2,3]. These techniques facilitate endovascular navigation and decrease the dose of radiation and the need for intravenous contrast agents by superimposing the patient’s preoperative 3D anatomy over the intraoperative 2D fluoroscopy [4,5]. Similarly, image guidance techniques could also be helpful in recently developing vascular robotic-assisted procedures, facilitating a focused approach towards the target vasculature while minimizing the unnecessary manipulation of the soft tissue. This could be achieved with augmented reality (AR), which overlays the patient’s individualized anatomy or preplanned surgical trajectories, derived from preoperative CT scans, directly onto the surgeon’s field of view.

Imaging-based AR navigation systems have been utilized in various surgical fields from spine surgery to urology and liver surgery, although these have stayed mostly experimental in application [6,7,8,9,10]. There have been reports of AR used in cardiothoracic procedures, although applications were mostly limited to endovascular procedures and the localization of lung nodules and lymph nodes during thoracic surgical procedures [11]. Evolving robotic vascular surgical procedures would also potentially benefit from such image-guidance systems due to the challenge of localizing small vasculature in the retroperitoneum without extensive dissection. This integration of imaging during robotic procedures could also potentially optimize the port placement and robot setup and minimize the risk of nerve damage or inadvertent vascular injury, thus increasing patient safety.

Our motivation for this study stems from various robotic vascular procedures where we used preoperative cinematically rendered CT images and compared them to the view from the endoscope in real-time to improve target localization intraoperatively [12,13]. We found this technique promising; however, the integration using AR was lacking. The nature of the robotic surgical equipment, namely the 3D image from the endoscope, and that the surgeons are able to interact directly with the computer, make it an ideal platform to develop an integrated, CT-based, AR navigation system [8].

Currently, one of the main challenges limiting the seamless integration of preoperative imaging such as CT for target localization during robotic vascular procedures is the physical displacement of tissue/target vessels due to factors such as patient positioning (PP) and elevated intra-abdominal pressure (IAP) due to CO_2_ (Carbon dioxide) gas insufflation compared to the patient’s preoperative scan, resulting in registration errors. However, there have been experiments directed to address this challenge in other surgical specialties [14,15,16]. Another confounding factor is the extent of surgical manipulation, namely retraction and pulling, which will have to be addressed in the future.

While organs in the intraperitoneal space move relatively freely under passive effects, we hypothesized that retroperitoneal structures are relatively stable in position due to the surrounding connective tissue. This would be ideal for the development of an accurate imaging-based guidance system; however, the quantitative data to address this hypothesis are lacking. Therefore, in this pilot study, we specifically sought to quantify the displacement of retroperitoneal target organs and vasculature due to abdominal CO_2_ insufflation and patient positioning.

## 2. Materials and Methods

**Figure 1:**
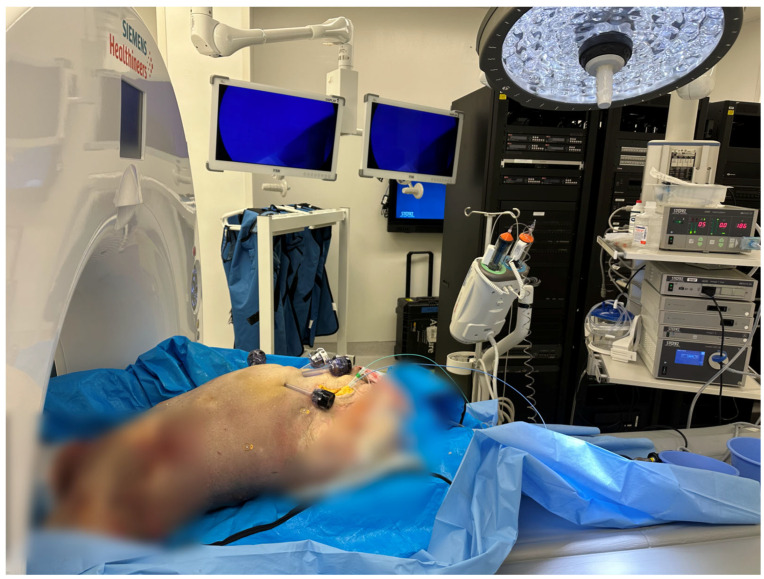
Example of the experimental setup. The cadaver is placed in Trendelenburg position; the intra-abdominal pressure is set at 5 mmHg.

The experiment was performed on a fresh frozen human cadaver at room temperature, around 20 °C. The cadaver was of an anonymous 88-year-old Caucasian male, with a normal BMI, who died of a cardiopulmonary cause and whose body was used 78 days after his death (Figure 1).

The right common femoral artery and vein were exposed through a surgical cut-down. A transverse arteriotomy was made, a 7 Fr 11 cm introducer sheath (Cordis, Miami Lakes, FL, USA) was placed in the common femoral artery and a 6 Fr 11 cm introducer sheath was placed in the common femoral vein. We then flushed the aorta and the inferior vena cava using a 5 Fr 65 cm long pigtail catheter (Cook Medical, Bloomington, IN, USA) with approximately 3 L of 0.9% saline solution to remove any cadaver clots and fill up the vessels with saline, followed by power injection of a large bolus (~100 mL) of iodinated contrast agent (320 mg/mL Visipaque^TM^, GE Healthcare, WI, USA) from the aortic arch to bifurcation. After inserting two 12 mm and two 5 mm laparoscopic ports into the abdominal wall, CT imaging was performed using the Vascular Angio Standard Routine settings on the Siemens Somatom Edge Dual Energy 164 Slice Scanner (Siemens Healthineers, Erlangen, Germany) with 0, 5, 15, and 25 mmHg intra-abdominal pressures in the supine, 45 degrees left lateral decubitus, 45 degrees right lateral decubitus, 15 degrees Trendelenburg, and reverse Trendelenburg positions, respectively. Acquisition parameters included a collimation of 0.6 mm × 34.8 mm, pitch 1.2, tube voltage 100 kVp, current 256 mAs, and dose–length product (DLP) 64.46 mGy·cm. Images were reconstructed at a 1 mm slice thickness. The patient’s positioning was stabilized using solid foam wedges (Table 1).

The reconstructed CT images were loaded into an open-source image processing software (3D Slicer, version 5.6.2. www.slicer.org), where multiplanar and 3D reconstructions were created. The fixed anatomical landmarks listed in Table 1 were marked with virtual fiducials in each scan. These were used as the reference matrix.

To be able to measure the displacement in the more peripheral tissue, we elected to mark the right and left hila of the kidneys at the point where the renal artery divides into segmental branches. We used virtual fiducials as markings in each scan.

**Figure 2:**
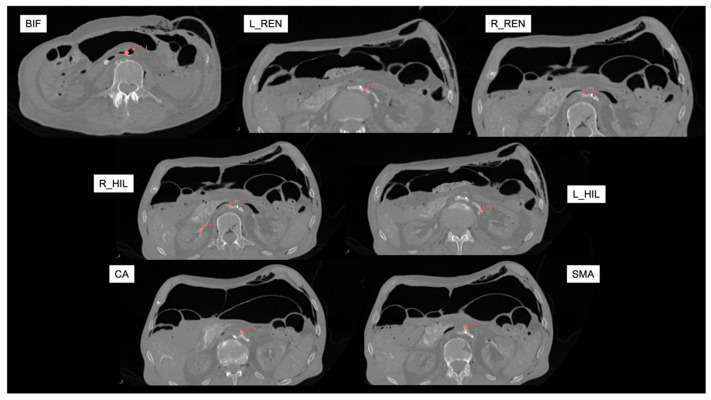
Reference axial CT angiography images (in supine position with zero mmHg intra-abdominal pressure) for marking targets. Red virtual fiducials were manually placed to mark each target landmark. BIF: aortic bifurcation; L_REN: left renal artery; R_REN: right renal artery; L_HIL: left hilum; R_HIL: right hilum; CA: Celiac artery; SMA: Superior Mesenteric artery.

The distal tip of the sternum was also used as a reference point, but later, it was excluded from the analysis due to its broad movement associated with abdominal expansion during IAP increase. Likewise, skin fiducials were used on the abdomen with a plan to use them as reference points, but their movement between patient positioning rendered them useless. This highlights the challenge associated with fiducial marker-based registration approaches in the surgical workflow. The other reference points used remained stationary across scans (Figure 2).

Distances between the anatomical reference landmarks and targets were measured using an open-label extension of 3D Slicer, called “Fiducials To Model Distance”. The measured Euclidean distances then were compared to each other between scans with different pressures/positions. All the scans performed in the same PP with different IAPs were compared to reveal the effect of IAP (base was zero mmHg). Then, all the scans with the same IAP but different positions were compared to show the effect of PP (the base was the supine position).

**Figure 3:**
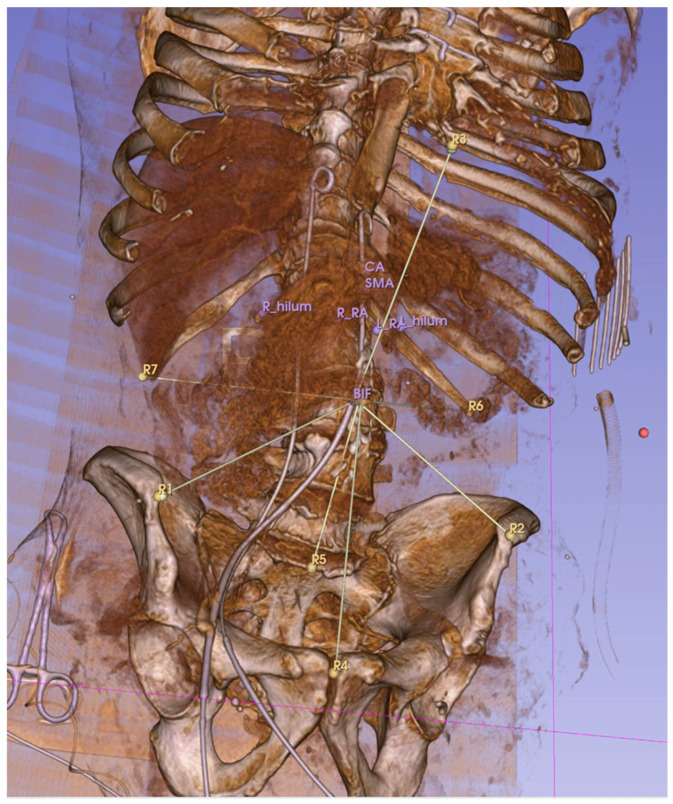
Euclidean distances between reference points (R1–R7) and target anatomical locations were measured. This 3D, reconstructed CT image shows the measurement lines connecting the aortic bifurcation to the reference landmarks R1–7. The distal tip of the sternum (R3 here) was later excluded due to its broad movement associated with the abdominal pressure increase.

Distances were calculated between each reference landmark and the target (see Figure 3). These distances were then compared between scans. For example, to calculate the effects of 15 mmHg IAP in the supine position, we measured the distance of targets to reference points in the CT scan done in the supine position at 0 mmHg and 15 mmHg. To measure the displacement of a target organ, we took the average of the absolute differences in distance to the reference point between the baseline scan (0 mmHg, supine position) and the one at 15 mmHg.

To determine the reproducibility of the marker placements in the software, the same operator repeated the placement of the virtual fiducials 3 times in one scan. Similarly, this method was used in another study [17].

This research utilized anonymized cadaveric specimens obtained through documented informed consent (including their use for research purposes) from donors or their legal representatives prior to death conducted by an outside contractor. This was done in compliance with all applicable legal and ethical standards for the use of human tissue in research, as well as the Declaration of Helsinki (1975, revised in 2013). Due to the use of a deidentified human cadaver, this research does not meet the regulatory definition of “human subject research”, and an IRB review was not required.

## 3. Results

A total of 20 contrast-enhanced CT scans were performed in different PPs and with different IAPs. The repositioning accuracy of virtual fiducials between different scans was an average of 2.8 mms (+/−1.2) in the positioning of the bone reference markers and 2.9 (+/−3.2) in anatomical target markers.

### 3.1. Effects of Intra-Abdominal Pressure

To determine the effects of intra-abdominal pressure with CO_2_ insufflation in each position, the cadaver was scanned in the same position with different IAPs from 0 to 25 mmHg. The result can be seen in Table 2. We found sub-one-centimeter changes in the average differences in distances from the reference landmarks, suggesting minimal movements due to the effects of raising the intra-abdominal pressure alone (Table 2).

### 3.2. Effects of Patient Position Change

To determine the effects of patient positioning, the cadaver was scanned in different PPs with the same IAPs (0–5–15–25 mmHg).

Apart from the hilum of the right kidney in the reverse Trendelenburg position at 25 mmHg of IAP, we experienced sub-one-centimeter differences in average distance changes compared to the reference points when changing the cadaver’s position (Table 3).

## 4. Discussion

In this study, we quantified the displacement of retroperitoneal anatomical landmarks in a cadaveric model under simulated laparoscopic conditions. We have compared the effects of positional changes and elevated IAP. The measured average displacements of target structures remained sub-centimetric with the increasing IAP, suggesting their relatively fixed position. However, positional changes induced slightly greater organ displacement, as evidenced by more substantial movement compared to pressure effects alone. It is important to note that surgical retraction, pulling, and dissection also have to be considered, but it is even more challenging to objectively measure these factors, as it most likely depends on the characteristics and elasticity of the tissue and the individual patient [18,19]. In theory, this could be accounted for using machine learning algorithms based on the tissue characteristics derived from imaging. To our knowledge, this is the first study to assess and quantify the deformation of retroperitoneal tissue and vasculature using CT imaging during laparoscopic procedures, while other studies examined other anatomical structures, mainly in the pelvis [17,20].

Our findings can provide valuable insights to enhance the registration accuracy of future preoperative, CT-based, intraoperative navigation systems. Image-guided surgery (IGS) holds the promise of improved target localization and patient safety, leading to individualized, “precision surgery” [21]. It has been previously proven to improve the outcomes of different surgical interventions, and it is even more relevant in robotic vascular surgery if we were to push the level of autonomy [22,23]. Despite its promising benefits, its broad applicability is theoretically hindered by the unknown factor of intraoperative organ or soft-tissue deformation compared to the preoperative 3D images. In addition, preoperative 3D imaging is also evolving with the advent of photon-counting CT, a dynamic, gated, time-resolved imaging technique that depicts the target vasculature more accurately, lending itself to optimized preoperative planning and intra-operative guidance for robotic surgery [24,25].

Laparoscopic and robotic methods have not been widely practiced by vascular surgeons. However, there is an abundance of vascular procedures performed mostly by other specialties [26]. Despite the better visibility in 3D images and the broad range of movements of wristed instruments, the lack of haptic feedback poses additional difficulties in the dissection of vascular structures. For example, the robotic or laparoscopic ligation of feeding vessels of a type II endoleak after EVAR requires the precise localization of these small branches in the retroperitoneum, in the vicinity of several nerves and other vascular structures prone to injury [27]. IGS would ideally be utilized in this field to decrease the need for extended and unneeded dissection. The latest generation of robots have evolved with better direct imaging, including stereoscopic and augmented image visualization that lends itself to an opportunity to integrate preoperative 3D anatomy during robotic vascular surgical procedures [28,29].

In this study, we simulated robotic laparoscopic procedures with relevant patient positioning scenarios used during specific vascular robotic procedures [26,30]. Regarding intra-abdominal pressures, most laparoscopic and robotic vascular procedures are carried out between 10–15 mmHg. High pressures, such as 25 mmHg, are unlikely and not part of standard practice. However, we wanted to evaluate tissue displacement at extremes of intra-abdominal pressure, with the validation of tissue deformation modeling in the back of our minds. There was a trend toward higher organ movement due to the use of higher IAPs, although the average reference-to-target discrepancy (e.g., displacement) between the scans did not exceed 1 cm.

Our study suggests that although there is deformation in the retroperitoneal target anatomy due to the aforementioned passive effects, it is likely relatively small, making it an ideal area to develop specific image-guided navigational systems.

Previous studies examined the clinical applicability of surgical navigation systems during laparoscopy. In a study focused on registration errors in laparoscopic gastrectomy, errors ranged from 2.1 to 32.9 mm. Despite the errors, participating surgeons found the system useful [31]. In liver surgery, the accuracy of the overlay using rigid registration can be expected to be around 10 mm; it can be decreased to about half of this using more complex, deformable registration [15]. Rigid registration models assume that the target organ is not deformed compared to the index imaging scan, while deformable registration models allow local transformations to account for anatomical changes between the reference scan and the live image. However, this comes at a price of increasing complexity.

Our preliminary findings demonstrated sub-centimetric average organ displacements, suggesting that such a system with non-deformable registration could be potentially utilized with acceptable results in the retroperitoneal space for vascular robotic procedures, although more testing involving living subjects is desired.

There are a few specific surgical navigation systems available that use AR to improve surgical accuracy [9]. One of which is the commercially available VisAR (Novarad, Provo, UT, USA), which has been utilized in spine and neurosurgery with good results [32,33]. A different AR-guided navigation reportedly minimized blood loss and improved the oncological prognosis in patients undergoing laparoscopic liver resection compared to indocyanine green fluorescence imaging [34].

Our study has several limitations that we need to address. First, the use of cadaveric tissue for the measurements, which possibly has different characteristics than live tissue, with different muscle tone and tissue properties. These cadaveric tissue attributes, namely the lack of perfusion and muscle tone, may have influenced our results; therefore, these findings may not be exactly transferable to living patients, warranting further studies [35]. However, ethical considerations due to the experiment’s invasiveness limit its feasibility in living human subjects. Nagaya et al. performed a similar study comparing organ position on CT scans in living human subjects performed in the supine and lateral positions, but only in preoperative scans without insufflation [36]. Intraoperative scans and simulating the effects of IAP are difficult to perform in living subjects. One possible solution would be the acquisition of a cone-beam CT in a hybrid operating room for patients undergoing laparoscopic procedures. Still, such scans’ quality in assessing small retroperitoneal targets raises concerns.

Our study’s second limitation is the lack of a fixed reference landmark matrix. We attempted to create this by our placement of radio-opaque fiducial markers (CT-Spot^®^, Beekley Medical, Bristol, CT, USA) on the body surface. This was more challenging than we anticipated in a surgical environment, both from a patient positioning standpoint and from a field sterility standpoint. Furthermore, these fiducials that were attached to the skin in various areas showed unpredictable movement with the PP and IAP changes. Thirdly, the use of only one cadaver to perform the experiments prevented us from comparing our findings across multiple subjects, which we plan to address in future studies, along with making the imaging data available to the imaging research community working on developing tissue deformation models and algorithms.

Despite these limitations, our study provides valuable insights into tissue displacement during laparoscopic procedures and serves as a base for future investigations, hopefully inspiring researchers to pursue the development of a CT imaging-based navigation tool to improve the precision of retroperitoneal dissections.

## 5. Conclusions

This study observed minimal displacement in retroperitoneal vascular structures across various intra-abdominal pressures with CO_2_ insufflation during simulated laparoscopic procedures. From the context of robotic vascular surgery, these data are encouraging for the development of further image guidance tools and techniques that could further be refined by real-time imaging techniques and advanced stereoscopic image visualization tools available in the latest generation of robotic systems. Further studies are needed to quantify the impact of tissue deformation on image guidance during various robotic vascular procedures to encourage the imaging research community to develop modeling tools for deformation compensation and to push the frontier of “true” image-guided robotic vascular surgery.

## Figures and Tables

**Table 1:** Reference points and targets used for measuring the displacement of retroperitoneal vasculature. * R7 was considered but was excluded due to the broad movement associated with abdominal expansion, limiting its usability as a fixed landmark.

Anatomical References		Anatomical Target Landmarks	
R1	Right Anterior Superior Iliac Spine	P1	Aortic Bifurcation
R2	Left Anterior Superior Iliac Spine	P2	Left Renal Artery
R3	Symphysis (upper edge)	P3	Right Renal Artery
R4	First Sacral Vertebra	P4	Hilum of the Left Kidney
R5	Tip of the Left XII. Rib	P5	Hilum of the Right Kidney
R6	Tip of the Right XII. Rib	P6	Superior Mesenteric Artery
R7 *	Distal Tip of the Sternum (excluded)	P7	Celiac Artery

**Table 2:** Average displacement of target anatomical points in mm (with standard deviation) compared to anatomical reference points at different pressures versus baseline (0 mmHg).

Target Anatomy	Pressure vs. Baseline (0 mmHg)	Supine	Right Lateral Decubitus	Left Lateral Decubitus	Trendelenburg	Reverse Trendelenburg
Aortic Bifurcation	5	0.5 (1.2)	0.8 (1.8)	0.1 (0.3)	0.7 (0.5)	0.8 (0.7)
	15	2.0 (1.3)	1.9 (2.5)	1.6 (1.1)	1.2 (1.2)	0.4 (0.5)
	25	1.7 (1.2)	1.8 (2.5)	2.5 (1.6)	2.3 (1.0)	2.2 (3.2)
Left Renal Artery	5	1.2 (1.2)	0.7 (1.3)	0.0 (0.0)	2.7 (1.8)	0.8 (0.5)
	15	1.2 (1.2)	2.8 (1.4)	1.3 (1.3)	2.7 (1.5)	0.8 (0.5)
	25	3.3 (2.9)	2.1 (1.6)	1.3 (1.3)	4.8 (1.2)	2.0 (2.2)
Right Renal Artery	5	3.7 (3.4)	0.7 (1.2)	0.2 (0.6)	1.7 (1.9)	1.0 (1.5)
	15	4.0 (3.2)	2.2 (0.9)	0.6 (0.6)	2.5 (2.4)	1.5 (2.4)
	25	3.5 (2.0)	1.9 (1.3)	0.9 (0.5)	5.5 (3.3)	2.4 (2.4)
Hilum of Left Kidney	5	0.7 (1.6)	0.6 (1.2)	0.3 (0.3)	1.2 (1.2)	0.8 (0.5)
	15	2.2 (1.9)	2.9 (1.4)	1.2 (0.9)	2.0 (0.6)	0.7 (0.5)
	25	2.0 (1.3)	2.0 (0.9)	1.7 (1.3)	2.5 (0.8)	2.1 (2.1)
Hilum of Right Kidney	5	1.2 (1.2)	0.6 (1.1)	0.4 (0.5)	3.0 (2.4)	2.9 (4.7)
	15	1.3 (1.4)	2.1 (1.9)	1.0 (0.7)	3.0 (1.5)	3.4 (4.6)
	25	2.5 (3.1)	2.1 (1.6)	0.8 (0.6)	4.2 (2.9)	3.8 (3.3)
Superior Mesenteric Artery	5	0.7 (1.6)	0.6 (1.1)	0.4 (0.6)	1.5 (1.2)	0.7 (0.5)
	15	2.2 (1.9)	2.7 (1.0)	0.8 (0.3)	1.5 (2.3)	1.2 (1.2)
	25	2.7 (1.8)	1.5 (1.4)	2.1 (1.3)	2.3 (1.0)	2.7 (3.0)
Celiac Artery	5	0.7 (1.6)	0.5 (1.0)	0.4 (0.7)	1.5 (2.0)	0.9 (0.8)
	15	1.5 (1.9)	2.7 (0.8)	0.6 (0.6)	2.3 (2.4)	1.2 (1.5)
	25	2.0 (1.1)	1.6 (1.3)	1.3 (1.0)	6.7 (2.6)	3.5 (3.5)

**Table 3:** Average displacement of target anatomical points in mm compared to anatomical reference points in different positions vs. baseline (supine).

Target Anatomy	Position vs. Baseline (Supine)	0 mmHg	5 mmHg	15 mmHg	25 mmHg
Aortic Bifurcation	Left lateral decubitus	4.1 (3.5)	4.2 (2.6)	3.3 (2.8)	4.6 (4.5)
	Right lateral decubitus	8.7 (3.4)	8.2 (3.8)	9.2 (4.8)	10.3 (4.7)
	Trendelenburg	4.9 (2.4)	4.4 (3.2)	5.0 (4.1)	3.5 (3.7)
	Reverse Trendelenburg	6.3 (4.8)	5.4 (5.0)	6.1 (5.3)	5.3 (3.7)
Left Renal Artery	Left lateral decubitus	4.0 (3.7)	5.2 (3.7)	6.5 (4.5)	7.4 (6.9)
	Right lateral decubitus	3.6 (3.6)	3.1 (2.7)	2.1 (1.9)	3.8 (3.2)
	Trendelenburg	4.0 (2.9)	1.6 (1.9)	1.7 (1.6)	4.2 (2.0)
	Reverse Trendelenburg	4.9 (4.3)	4.8 (4.0)	4.4 (4.2)	2.9 (2.7)
Right Renal Artery	Left lateral decubitus	8.0 (5.2)	6.0 (4.2)	6.2 (3.6)	7.1 (3.2)
	Right lateral decubitus	2.5 (1.0)	3.9 (2.5)	4.2 (1.7)	3.4 (1.9)
	Trendelenburg	3.7 (2.6)	2.8 (2.8)	2.4 (2.6)	4.8 (3.3)
	Reverse Trendelenburg	4.6 (4.4)	5.6 (4.7)	4.4 (5.0)	3.1 (3.8)
Hilum of Left Kidney	Left lateral decubitus	4.0 (1.8)	4.0 (2.1)	2.8 (1.3)	3.9 (2.0)
	Right lateral decubitus	2.9 (2.1)	2.5 (2.5)	2.8 (1.5)	2.8 (2.0)
	Trendelenburg	1.5 (1.0)	2.5 (1.0)	3.6 (1.9)	2.8 (2.1)
	Reverse Trendelenburg	5.8 (5.0)	5.7 (5.0)	6.6 (4.9)	5.7 (4.4)
Hilum of Right Kidney	Left lateral decubitus	6.7 (2.8)	6.5 (4.1)	5.4 (2.7)	4.2 (3.1)
	Right lateral decubitus	2.8 (1.5)	1.9 (1.3)	1.6 (1.6)	3.0 (3.8)
	Trendelenburg	2.1 (1.5)	3.0 (1.8)	1.9 (2.3)	4.0 (3.1)
	Reverse Trendelenburg	14.0 (6.3)	13.5 (6.5)	15.9 (8.3)	16.6 (8.9)
Superior Mesenteric Artery	Left lateral decubitus	5.8 (5.0)	5.7 (4.5)	6.0 (4.3)	6.5 (4.4)
	Right lateral decubitus	5.8 (5.2)	6.5 (5.0)	6.6 (4.1)	5.4 (3.7)
	Trendelenburg	3.1 (3.3)	4.2 (3.4)	4.5 (3.8)	3.5 (2.7)
	Reverse Trendelenburg	7.6 (6.2)	8.2 (5.8)	8.7 (6.2)	6.2 (5.4)
Celiac Artery	Left lateral decubitus	5.2 (2.4)	5.1 (3.1)	4.1 (3.2)	4.5 (3.0)
	Right lateral decubitus	3.6 (3.2)	4.6 (2.9)	5.5 (3.0)	3.8 (1.8)
	Trendelenburg	4.9 (3.3)	5.2 (3.4)	5.6 (4.0)	3.4 (2.0)
	Reverse Trendelenburg	6.4 (5.1)	6.6 (5.0)	7.1 (5.4)	4.1 (4.6)

## Data Availability

The study data (including the DICOM imaging data) are available upon reasonable request to the corresponding author.

## Abbreviations

The following abbreviations are used in this manuscript:

PP	patient position
IAP	intra-abdominal pressure
AR	augmented reality
CT	computer tomography
IGS	image-guided surgery
